
JOURNAL INFORMATION
==============================
NLM Title Abbreviation: Orthop Surg
Journal ID: Orthop Surg
NLM Journal ID: 101501666
Journal ID: OS

Orthopaedic Surgery

ISSN: 1757-7853
EISSN: 1757-7861
Publisher: Wiley

ARTICLE INFORMATION
==============================
PMCID: PMC11474544
DOI: 10.1111/os.13786
Article ID: OS13786
Article version: 1
Subjects: Issue Information, Issue Information

Issue Information

Electronic publication date: 2024 Jun 2
Publication date: 2024 Jun
Volume: 16
Issue: 6
First page: 1254
Last page: 1255
Copyright: © 2024 The Authors. Orthopaedic Surgery published by Tianjin Hospital and John Wiley & Sons Australia, Ltd.
License: This is an open access article under the terms of the http://creativecommons.org/licenses/by/4.0/ License, which permits use, distribution and reproduction in any medium, provided the original work is properly cited.
License URL: https://creativecommons.org/licenses/by/4.0/

Figure count: 0
Table count: 0
Page count: 12

source-schema-version-number: 2.0
cover-date: June 2024
details-of-publishers-convertor: Converter:WILEY_ML3GV2_TO_JATSPMC version:6.4.9 mode:remove_FC converted:15.10.2024

==============================

Volume 16 • Issue 6 • June 2024 • ISSN 1757-7861

### Orthopaedic Surgery

Open Access

REVIEW ARTICLE

1257
ADVANCES IN THE CAUSES AND TREATMENT OF FLOATING TOES AFTER THE WEIL OSTEOTOMY: A SCOPING REVIEW

CLINICAL ARTICLES

1269
DOES THE SAGITTAL RADIOGRAPHIC MORPHOLOGY OF SUBTALAR JOINT AFFECT THE ALIGNMENT OF FOOT?

1277
EFFICACY OF A NOVEL INTRAOPERATIVE SURGICAL IRRIGANT IN PREVENTING PERIPROSTHETIC JOINT INFECTIONS IN PRIMARY KNEE, HIP, AND SHOULDER ARTHROPLASTIES: A RETROSPECTIVE ANALYSIS

1284
RISK FACTORS FOR SURGICAL TREATMENT OF LUMBAR DEGENERATIVE DISC DISEASE IN MIDDLE-AGED AND OLDER WOMEN: A PROSPECTIVE CASE–CONTROL STUDY OF 2370 SUBJECTS

1292
CLINICAL ANALYSIS OF MICROWAVE ABLATION COMBINED WITH DECOMPRESSION AND PEDICLE SCREW FIXATION IN THE TREATMENT OF SPINAL METASTASES

1300
THE LONG-TERM EFFICACY OF TOTAL KNEE ARTHROPLASTY ON END-STAGE KASHIN–BECK DISEASE OF THE KNEE IN HIGHLAND TIBETAN AREAS PATIENTS: A RETROSPECTIVE STUDY WITH 10-YEAR FOLLOW-UP

1308
DO AGE AND TIMING INFLUENCE THE OUTCOMES OF SINGLE-STAGE RECONSTRUCTION OF MULTIPLE LIGAMENT KNEE INJURIES? 5-10 YEARS FOLLOW UP

1317
THE EFFICACY OF ANTERIOR CRUCIATE LIGAMENT RECONSTRUCTION WITH PERONEUS LONGUS TENDON AND ITS IMPACT ON ANKLE JOINT FUNCTION

1327
EFFECTS OF CAGE IMPLANTATION DEPTH ON SAGITTAL PARAMETERS AND FUNCTIONAL OUTCOMES IN POSTERIOR LUMBAR INTERBODY FUSION FOR THE TREATMENT OF L4-L5 LUMBAR DEGENERATIVE SPONDYLOLISTHESIS

1336
REPEAT SURGERY AFTER PERCUTANEOUS ENDOSCOPIC LUMBAR DISCECTOMY FOR ADOLESCENT LUMBAR DISC HERNIATION: A MULTICENTER OBSERVATIONAL STUDY

1344
THE CLINICAL APPLICATION OF DOUBLE TAYLOR SPATIAL FRAME IN SEGMENTAL TIBIAL FRACTURE

1356
THE INFLUENCE OF RADIOGRAPHIC PARAMETER ON THE S2 ALAR-ILIAC SCREW VIRTUAL TRAJECTORY IN DEGENERATIVE LUMBAR SCOLIOSIS PATIENTS: A COMPUTED TOMOGRAPHY STUDY

1364
CHRONIC ACLD KNEES WITH EARLY DEVELOPMENTAL CARTILAGE LESIONS EXHIBITED INCREASED POSTERIOR TIBIAL TRANSLATION DURING LEVEL WALKING

1374
CLINICAL STUDY OF A FOUR-STEP PROGRAM FOR THE TREATMENT OF PLANTAR FASCIITIS WITH BONE SPURS

1381
DEVELOPING A MACHINE-LEARNING PREDICTIVE MODEL FOR RETENTION OF POSTERIOR CRUCIATE LIGAMENT IN PATIENTS UNDERGOING TOTAL KNEE ARTHROPLASTY

1390
HYPER-SELECTIVE POSTERIOR FUSION IS RECOMMENDED WHEN THE MODIFIED S-LINE IS POSITIVE IN LENKE 5C ADOLESCENT IDIOPATHIC SCOLIOSIS

1399
MID-TERM CLINICAL OUTCOMES OF “LIGHT BULB” CORE DECOMPRESSION WITH ARTHROSCOPIC ASSISTANCE IN PERI-COLLAPSE OSTEONECROSIS OF THE FEMORAL HEAD: A RETROSPECTIVE COMPARATIVE STUDY

1407
DOES FOCAL KYPHOTIC DEFORMITY AT NON-RESPONSIBLE LEVELS AFFECT THE OUTCOMES OF ANTERIOR CERVICAL DECOMPRESSION AND FUSION?

RESEARCH ARTICLES

1418
UNRAVELING KEY $M^6A$ MODIFICATION REGULATORS SIGNATURES IN POSTMENOPAUSAL OSTEOPOROSIS THROUGH BIOINFORMATICS AND EXPERIMENTAL VERIFICATION

1434
CAN ROBOTIC ARM-ASSISTED TOTAL KNEE ARTHROPLASTY REMAIN COST-EFFECTIVE IN VOLUME-BASED PROCUREMENT SYSTEM IN CHINA? A MARKOV MODEL-BASED STUDY

1445
PREPARATION OF AG@3D-$TIO_2$ SCAFFOLDS AND DETERMINATION OF ITS ANTIMICROBIAL PROPERTIES AND OSTEOGENESIS-PROMOTING ABILITY

1461
EFFECT OF INSERTIONAL DIRECTION OF PEDICLE SCREW ON SCREW LOOSENING: A BIOMECHANICAL STUDY ON SYNTHETIC BONE VERTEBRA UNDER A PHYSIOLOGY-LIKE LOAD

OPERATIVE TECHNIQUES

1473
A JOY-STICK ASSISTANT THREE-DIMENSIONAL MODIFIED TECHNIQUE OF MINIMALLY INVASIVE SURGERY FOR MILD OR MODERATE HALLUX VALGUS

1480
FULL-ENDOSCOPIC TRANSFORAMINAL DEBRIDEMENT AND DECOMPRESSION FOR BRUCELLAR THORACIC SPINAL EPIDURAL ABSCESS: A MINIMALLY INVASIVE ALTERNATIVE TO OPEN SURGERY

1487
3D-PRINTED MULTIFUNCTIONAL GUIDE PLATE FOR FENESTRATION AND SCREWS DRILL IN PROXIMAL FEMORAL BENIGN TUMOR

1493
TREATMENT OF COMPLEX CENTRAL TALAR FRACTURES WITH LATERAL PLATE COMBINATION WITH MEDIAL SCREW FIXATION

BRIEF REPORT

1502
OSSIFICATION OF BILATERAL SACROTUBEROUS LIGAMENTS: TWO CASES REPORT AND LITERATURE REVIEW

CASE REPORT

1508
USING 3D PRINTING TECHNOLOGY TO DESIGN SPLIT-PIECE SLEEVE PROSTHESIS IN THE REVISION SURGERY OF TUMOR-TYPE TOTAL ELBOW PROSTHETIC FRACTURES: A CASE REPORT

WILEY

**View this journal online at wileyonlinelibrary.com/journal/os**

### Orthopaedic Surgery

**Honorary Editor-in-Chief**
Guixing Qiu, *Beijing, China*

**Editor-in-Chief**
Xinlong Ma, *Tianjin, China*

**Associate Editors**
Cyrus Cooper, *Southampton, UK*
Benjamin G. Domb, *Des Plaines, USA*
Qing Jiang, *Nanjing, China*
Baoge Liu, *Beijing, China*
Zhuojing Luo, *Xi'an, China*
Luigi Meccariello, *Moiano, Italy*
Ling Qin, *Hong Kong, China*
Qi Yin, *Liverpool, UK*

**Editorial Board**
Elisa Belluzzi, *Padua, Italy*
Carlo Biz, *Padua, Italy*
Mary L Bouxsein, *Boston, USA*
Richard E. Buckley, *Calgary, Canada*
Zhengdong Cai, *Shanghai, China*
Wei Chai, *Beijing, China*
Bohua Chen, *Qingdao, China*
Liang Chen, *Soochow, China*
Yanxi Chen, *Shanghai, China*
Pramod Devkota, *Patan, Nepal*
Meng Fan, *Tianjin, China*
Haoyu Feng, *Taiyuan, China*
Maoqi Gong, *Beijing, China*
Yong Hai, *Beijing, China*
Wei Huang, *Chongqing, China*
Baochao Ji, *Urumqi, China*
Ayhan Kanat, *Rize, Turkey*
Kenny Kwan, *Hong Kong, China*
Sallie Lamb, *Exeter, UK*
Lifeng Lao, *Shanghai, China*
Jung-Hee Lee, *Seoul, Korea*
Guanghua Lei, *Changsha, China*
Guoan Li, *Newton, USA*
Hui Li, *Tianjin, China*
Jianmin Li, *Jinan, China*
Weishi Li, *Beijing, China*
Jianhao Lin, *Beijing, China*
Fanxiao Liu, *Munich, Germany*
Ruben Lostado, *La Rioja, Spain*
Songchen Lyu, *Harbin, China*
Weihu Ma, *Ningbo, China*
Xin Ma, *Shanghai, China*
Masaya Nakamura, *Tokyo, Japan*
Muthu Sathish, *Velayuthampalayam, India*
Wolfgang Senker, *Linz, Austria*
Sancar Serbest, *Boston, USA*
Cailiang Shen, *Hefei, China*
Bin Shen, *Chengdu, China*
Huiyong Shen, *Shenzhen, China*
Jianxiong Shen, *Beijing, China*
Puyi Sheng, *Guangzhou, China*
Junying Sun, *Soochow, China*
Shui Sun, *Jinan, China*
YasuhikoTakegami, *Nagoya, Japan*
Sakae Tanaka, *Tokyo, Japan*
Xiaodong Tang, *Beijing, China*
Fei Wang, *Shijiazhuang, China*
Weijun Wang, *Nanjing, China*
Xu Wang, *Shanghai, China*
Zheng Wang, *Beijing, China*
Jidapa Wongcharoenwatana, *Bangkok, Thailand*
Chi-Chuan Wu, *Taipei, China*
Nan Wu, *Beijing, China*
Zhihong Wu, *Beijing, China*
Yayi Xia, *Lanzhou, China*
Jianru Xiao, *Shanghai, China*
Zhongyu Xie, *Shenzhen, China*
Dan Xing, *Beijing, China*
Weiguo Xu, *Tianjin, China*
Peng Xu, *Xi 'an, China*
CaoYang, *Wuhan, China*
Pei Yang, *Xi 'an, China*
Zhaoming Ye, *Hangzhou, China*
Xiuchun Yu, *Jinan, China*
Bin Yu, *Guangzhou, China*
Yi Zeng, *Chengdu, China*
Dianying Zhang, *Beijing, China*
Hong Zhang, *Texas, USA*
Weibin Zhang, *Shanghai, China*
Xianlong Zhang, *Shanghai, China*
Jianguo Zhang, *Beijing, China*
Bin Zhao, *Taiyuan, China*
Jie Zhao, *Shanghai, China*
Yu Zhao, *Beijing, China*
Zongke Zhou, *Chengdu, China*
Yue Zhu, *Shenyang, China*
Zezhang Zhu, *Nanjing, China*

**Address for Editorial Correspondence:** Editorial Board of *Orthopaedic Surgery*, Tianjin Hospital, 122 Munan Road, Tianjin 300050, China (email: orthopaedicsurgery@126.com; telephone: +86 22 2833 4734; fax: +86 22 2824 1184).

**Disclaimer:** The Publisher, Tianjin Hospital and Editors cannot be held responsible for errors or any consequences arising from the use of information contained in this journal; the views and opinions expressed do not necessarily reflect those of the Publisher, Tianjin Hospital and Editors, neither does the publication of advertisements constitute any endorsement by the Publisher, Tianjin Hospital and Editors of the products advertised.

**Copyright © 2024 The Authors. Orthopaedic Surgery published by Tianjin Hospital and John Wiley & Sons Australia, Ltd**

**Copyright and Copying (in any format)**
Copyright © 2024 The Authors. Orthopaedic Surgery published by Tianjin Hospital and John Wiley & Sons Australia, Ltd. All rights reserved. No part of this publication may be reproduced, stored or transmitted in any form or by any means without the prior permission in writing from the copyright holder. Authorization to copy items for internal and personal use is granted by the copyright holder for libraries and other users registered with their local Reproduction Rights Organisation (RRO), e.g. Copyright Clearance Center (CCC), 222 Rosewood Drive, Danvers, MA 01923, USA (www.copyright.com), provided the appropriate fee is paid directly to the RRO. This consent does not extend to other kinds of copying such as copying for general distribution, for advertising or promotional purposes, for republication, for creating new collective works or for resale. Permissions for such reuse can be obtained using the RightsLink "Request Permissions" link on Wiley Online Library. Special requests should be addressed to: permissions@wiley.com

For submission instructions, subscription and all other information visit: http://wileyonlinelibrary.com/journal/os

ISSN 1757-7861 (Online)

OS.JEB.June24
